# Naïve T Cell Homeostasis Regulated by Stress Responses and TCR Signaling

**DOI:** 10.3389/fimmu.2015.00638

**Published:** 2015-12-17

**Authors:** Daisuke Kamimura, Toru Atsumi, Andrea Stofkova, Naoki Nishikawa, Takuto Ohki, Hironao Suzuki, Kokichi Katsunuma, Jing-jing Jiang, Hidenori Bando, Jie Meng, Lavannya Sabharwal, Hideki Ogura, Toshio Hirano, Yasunobu Arima, Masaaki Murakami

**Affiliations:** ^1^Division of Molecular Neuroimmunology, Institute for Genomic Medicine, Graduate School of Medicine, Hokkaido University, Sapporo, Japan; ^2^Laboratory of Developmental Immunology, WPI Immunology Frontier Research Center, Graduate School of Frontier Biosciences, Osaka University, Suita, Japan; ^3^Laboratory of Developmental Immunology, WPI Immunology Frontier Research Center, Graduate School of Medicine, Osaka University, Suita, Japan; ^4^Osaka University, Suita, Japan

**Keywords:** T cell homeostasis, stress response, TCR signaling, naive T cells, mutagenesis

## Abstract

The survival of naïve T cells is believed to require signals from TCR–pMHC interactions and cytokines such as IL-7. In contrast, signals that negatively impact naïve T cell survival are less understood. We conducted a forward genetic screening of mice and found a mutant mouse line with reduced number of naïve T cells (T-Red mice). T-Red mice have a point mutation in the Kdelr1 gene, and their naïve T cells show enhanced integrated stress response (ISR), which eventually induces their apoptosis. Therefore, naïve T cells require a KDEL receptor-mediated mechanism that efficiently relieves cellular stress for their survival *in vivo*. Interestingly, naïve T cells expressing TCR with higher affinity/avidity to self-antigens survive in T-Red mice, suggesting the possible link between TCR-mediated survival and ISR-induced apoptosis. In this article, we discuss the regulation of naïve T cell homeostasis, keeping special attention on the ISR and TCR signal.

## Positive Signals for T Cell Homeostasis: Lessons from Previous Studies

T cell numbers in the periphery are almost constant, even though millions of naïve T cells are generated from the thymus daily. This homeostasis likely requires strict regulation. Additionally, naïve T cells have a relatively long half-life of approximately >50 days, and memory T cells show basal turnover to survive longer in the periphery (1–3). Survival signals for naïve T cells include TCR–pMHC interactions and cytokines such as IL-7, whereas the homeostasis of memory T cells is largely dependent on cytokines such as IL-2 and IL-15 along with IL-7 (4).

When peripheral naïve and memory T cells are decreased due to involution of the thymus by aging, infection, or irradiation, the remaining T cells start to proliferate, a phenomenon called homeostatic proliferation. At the molecular level, homeostatic proliferation is induced by TCR signaling and/or cytokines (5). Homeostatic proliferation sometimes plays a role in the activation of autoreactive T cells, causing several autoimmune diseases (6, 7). This homeostatic proliferation can be experimentally induced by a sublethal dose of irradiation in mice called lymphopenia-induced proliferation (LIP). LIP has greatly contributed to the understanding of the mechanism of T cell homeostasis *in vivo*, which otherwise takes a long time to study due to the slow turnover of these cells. LIP of naïve CD4^+^ and CD8^+^ T cells does not occur in the absence of TCR–pMHC interactions or IL-7 (5, 8), and genetic deficiency of IL-7 in host animals abrogates LIP (9). Compared to the involvement of cytokines, the contribution of TCR signaling to T cell homeostasis is more complicated. Homeostatic proliferation induced by TCR–pMHC interactions involves clonal competition among T cells, because LIP of monoclonal TCR transgenic (Tg) T cells is inhibited in TCR Tg mice that have the same monoclonal TCR (10–13). These observations suggest that not all T cells equally proliferate under LIP; those that have higher TCR affinity to self-antigens receive more TCR signaling to outcompete T cells expressing TCR with lower self-affinity in the periphery. Measurement of TCR avidity to self-peptide–MHC complexes under normal polyclonal conditions is challenging. However, it is known that CD5 can be a surrogate marker that reports the TCR signal strength in T cells. CD5 is a negative regulator of TCR signaling and whose expression correlates with the avidity of TCR (14, 15). Surface CD5 levels in T cells positively correlate with the phosphorylation levels of the CD3ζ chain, which is a proximal signal event after TCR ligation (15), and with Nur77 expression, which is an early target gene of TCR signaling (16). Indeed, LIP of CD8^+^ T cells derived from TCR Tg mice with high CD5 expression, such as OT-I cells, is faster than that of CD8^+^ T cells derived from other TCR Tg mice with lower CD5 expression (17). Thus, TCR affinity to self-pMHC contributes to the degree of survival signals that T cells receive. It is known that IL-7-mediated cell death is suppressed in CD8^+^ T cells by TCR signaling via self-pMHC. For example, IL-7Rα expression on T cells bearing low-affinity TCR requires TGF-β signaling (18). Continuous IL-7 signaling triggers cytokine-induced cell death in CD8^+^ T cells, while homeostatic engagement of TCR interrupts IL-7-mediated cell death. Consistent with these properties, CD8^+^ T cells with insufficient TCR affinity via self-pMHC induce IL-7-mediated cell death (19). These studies clearly suggest that T cell survival is tightly regulated by the TCR signal via self-pMHC and cytokines *in vivo*.

## T-Red Mice: Mutant Animals with Enhanced Naïve T Cell Death

To obtain more insight into T cell survival and death, we conducted an *N*-ethyl-*N*-nitrosourea (ENU) mutagenesis experiment and searched for mutant mice with unusual naïve vs. memory phenotype T cell ratios in peripheral blood. After the establishment of a mutant line that has excess CD44^High^ memory-phenotype T cells in peripheral blood (20, 21), we enumerated T cell numbers in the spleen and lymph nodes, revealing that CD44^High^ memory-phenotype T cells were not increased, but rather CD44^Lo^ naïve T cells were significantly decreased. Intriguingly, the phenotype of cellular loss was selective to naïve T cells because other immune cells, such as γδT cells, neutrophils, and dendritic cells, were not significantly reduced. Therefore, we named this mutant T-Red (naïve T cell reducing) mice (21). Compared to WT naïve T cells, the survival of T-Red naïve T cells is impaired *in vitro* and *in vivo*. Several T cell-dependent immune responses, such as collagen-induced arthritis and T cell responses to bacterial infection, are severely diminished in T-Red mice (21). Importantly, this mutant mouse is suitable for the investigation of naïve T-cell homeostasis *in vivo*.

## KDEL Receptor 1 (Kdelr1) is the Gene Responsible for T-Red Mice

To identify the gene responsible for the ENU mutants, T-Red mice (C57BL/6 background) were crossed with mice on a different genetic background (C3H/He). The resulting F1 hybrids were intercrossed, and the CD44^High^ T-Red phenotype was screened out in the F2 generation. Positional cloning using SNP markers detecting C57BL/6 genomic regions and DNA sequence analysis of T-Red mice on the mixed background identified an amino acid substituting point mutation (S123P) in the gene encoding KDEL receptor 1 (Kdelr1) (21). It is reported that the mutated residue, serine 123, of Kdelr1 is involved in the receptor’s conformation (22), suggesting that the T-Red mutation causes dysfunction of Kdelr1. Because multiple mutations are inevitably induced in the ENU mutant, a rescue experiment and generation of Kdelr1-deficient mice including CD4 T cell-specific Kdelr1 KO mice were performed, formally demonstrating that Kdelr1 is the responsible gene for the T-Red phenotype (21).

**Figure 1:**
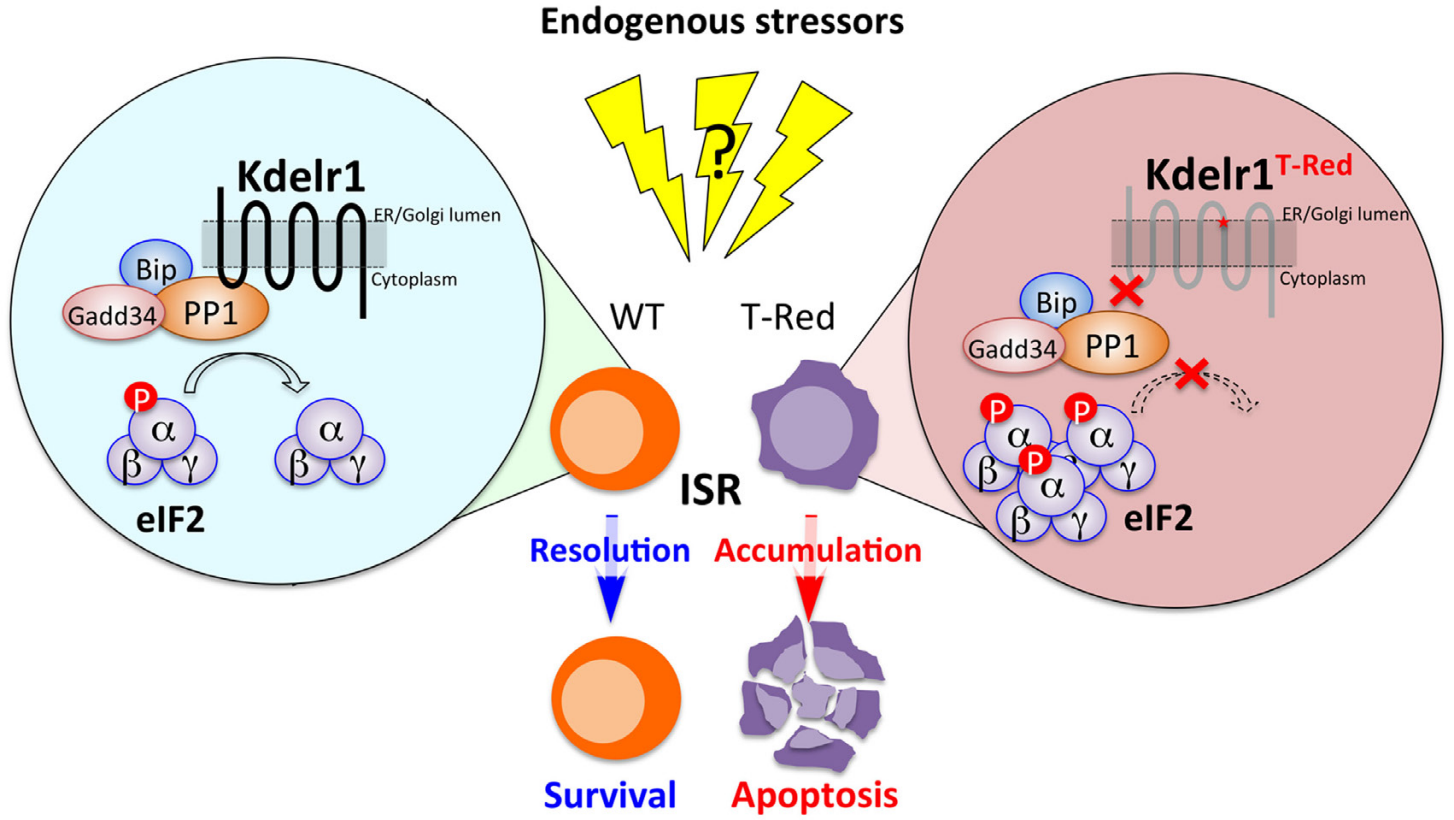
**The Kdelr1–PP1 axis is required for naïve T cell survival**. In WT naïve T cells, PP1 associated with Kdelr1 exhibits optimal phosphatase activity to prevent unwanted persistence of ISR. In T-Red naïve T cells, however, Kdelr1 dysfunction results in reduced PP1 activity, leading to prolonged ISR and apoptosis. Endogenous factors that cause ISR *in vivo* remain unidentified.

The KDEL receptor was originally identified as a chaperone retrieval receptor that recovers soluble endoplasmic reticulum (ER)-resident chaperones from the cis-Golgi. This Golgi-to-ER retrograde transport requires the binding of KDEL receptors with a KDEL motif localized at the C-terminal of the ER chaperones (23–25). Examples of actual and potential KDEL receptor ligands having the KDEL motif or its variants include Bip (Grp78), calreticulin, Hsp90b1, and several FK506-binding proteins (26). In mammals, there are three KDEL receptors, Kdelr1, Kdelr2, and Kdelr3, all of which are localized around ER and Golgi (27). A role for KDEL receptors beyond chaperone retrieval was also reported, as chaperone-bound KDEL receptors trigger the activation of Src family kinases at the Golgi complex to generate intracellular signaling cascades that coordinate the secretory pathway (27, 28). Furthermore, consistent with their ER-Golgi localization, KDEL receptors are involved in ER stress responses (29). Using T-Red mice, we found a novel role for Kdelr1 in naïve T cell homeostasis (Figure 1).

## Kdelr1–PP1 Axis: A New Mechanism That Alleviates Cellular Stresses in Naïve T Cells

Transcriptome analysis provided a clue about how Kdelr1 regulates the survival of naïve T cells. In T-Red naïve CD4^+^ and CD8^+^ T cells, gene expressions related to the integrated stress response (ISR), including those of Asns (asparagine synthetase), Trib3, Chop, and Vegfa, were significantly increased. The expression of Bim, which is a proapoptotic factor critically involved in T cell death, is controlled by Chop (30), suggesting that stress increases Bim expression in T-Red mice. Consistent with this notion, Bim levels are elevated in T-Red mice.

**Figure 2:**
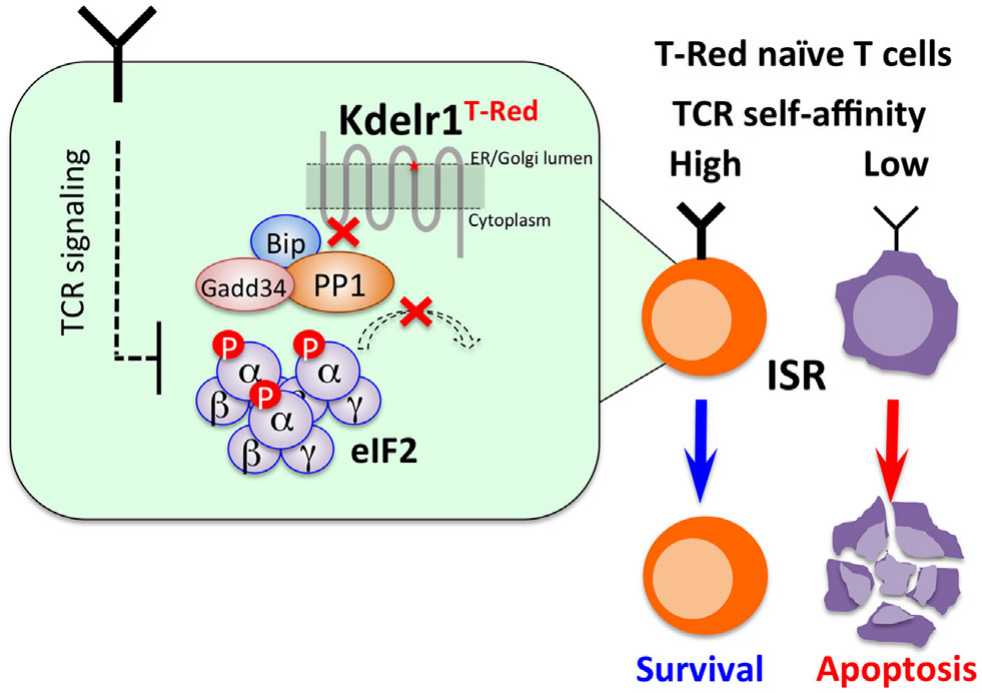
**Strong TCR-mediated signals alleviate ISR in T-Red naïve T cells**. T-Red naïve T cells expressing TCR with relatively high self-affinity are resistant to enhanced ISR caused by Kdelr1 dysfunction. Further studies are required to reveal the specific TCR signaling that counteracts ISR.

Integrated stress response is a cellular response that is induced by various types of stress signals, including ER stress, amino acid deprivation, infection with double-stranded RNA viruses, heme deficiency, and oxidative stress. All these stressors increase the phosphorylation at serine 51 of the α subunit of eukaryotic initiation factor 2 (eIF2α). Thus, eIF2α phosphorylation is a key event to triggering ISR (31–34). Indeed, T-Red naïve T cells have excess phosphorylation of eIF2α (21). We therefore considered how the excessive phosphorylation of eIF2α is regulated. It has been reported that phosphorylation of eIF2α is mediated by four kinases: double-stranded RNA-dependent protein kinase R (PKR), RNA-dependent protein kinase-like ER kinase (PERK), general control non-repressed 2 (GCN2), and heme-regulated eIF2α kinase (HRI) (35). However, the activation status of these four kinases was not enhanced in T-Red naïve T cells, suggesting that Kdelr1 is not involved in their regulation. The phosphorylated form of eIF2α is efficiently dephosphorylated by protein phosphatase 1 (PP1) to complete ISR. Importantly, PP1 activity in T-Red naïve T cells was reduced compared with WT naïve T cells. Additionally, the phosphorylation of eIF2α is evident in freshly isolated WT naïve T cells, suggesting the existence of certain stressor(s) for naïve T cells *in vivo* (21). Moreover, Kdelr1 associated with PP1, whereas mutant Kdelr1 did not. These results suggested that Kdelr1 is required for optimal PP1 activity to dephosphorylate eIF2α in naïve T cells under stress. Thus, the Kdelr1–PP1 axis regulates naïve T cell death, which is induced by ISR *in vivo* (Figure 2) (21).

## Strong TCR-Mediated Signals Alleviate ISR *in vivo*

As described above, T-Red mice show the increased CD44^High^ phenotype in T cells due to a reduction of naïve T cell numbers. Interestingly, the CD44^High^ phenotype became normal when T-Red mice were bred with TCR Tg mice such as OT-I and P14 mice. Absolute cell numbers of TCR Tg T cells were also the same between T-Red/TCR Tg and control TCR Tg mice (21). OT-I TCR Tg mice have TCR, which recognizes an ovalbumin (OVA) peptide on MHC class I-restricted TCR, and P14 TCR Tg mice express TCR, which recognize a lymphocytic choriomeningitis virus peptide on MHC class I, although specific endogenous antigen peptides are not known in either TCR Tg cases. However, it is known that both transgenic TCRs have relatively high self-peptide affinity (16, 17). Therefore, we hypothesized that naïve T cells that receive relatively stronger TCR signals due to higher self-affinity might be resistant to enhanced ISR. Consistent with this idea, the surviving naïve T cells in T-Red mice express significantly higher levels of CD5 (14, 15). In addition, a tetramer dissociation assay using peptides derived from self-antigens confirmed higher TCR affinity/avidity in T-Red naïve T cells. These results suggest a novel link between TCR signal strength and ISR-mediated apoptosis during steady state in naïve T cells.

These findings prompted us to examine the possibility of whether TCR stimulations with a panel of peptide ligands having various affinities to TCR may result in different responses between WT and T-Red naïve T cells *in vivo* and *in vitro*. We used ovalbumin (OVA)-altered peptide ligands (APL) as a model antigen (36–38) to stimulate control OT-I and T-Red/OT-I T cells and assessed the survival response *in vitro* and proliferation response *in vivo*. We found that weak TCR stimulation decreases the survival and proliferation of T-Red naïve T cells, whereas stimulation with ligands having higher affinity has no such effect (39). These results suggest that a strong physiological TCR signal suppresses IRS, whereas a weak one does not (Figure 2).

## Future Directions

Even in steady state, *in vivo* T cells have a certain level of eIF2α phosphorylation, suggesting that they receive a certain level of stress. However, the endogenous stressors for T cells particularly *in vivo* are unknown. Because the thymus from mice deficient in PERK has reduced levels of eIF2α phosphorylation, at least in the pancreas (40), it might be possible that some activators of eIF2α kinases are stressors in naïve T cells. In summary, we identified that the Kdelr1–PP1 axis plays a beneficial role for naïve T cells to resolve cellular stress *in vivo*. Our study also revealed that TCR signaling can counteract ISR in naïve T cells. A molecular mechanism behind TCR signaling and the resistance to ISR should be elucidated in the near future.

## Author Contributions

All authors contributed to write and elaborate the manuscript.

## Conflict of Interest Statement

The authors declare that the research was conducted in the absence of any commercial or financial relationships that could be construed as a potential conflict of interest.
